# ‘Days of Frustration’: A Qualitative Study of Adolescents’ Thoughts and Experiences of Schooling after Early Dropout

**DOI:** 10.3390/bs13110894

**Published:** 2023-10-29

**Authors:** Karl Ottar Ottosen, Charlotte Bjørnskov Goll, Rolf Wynn, Tore Sørlie

**Affiliations:** 1 Department of Clinical Medicine, UiT The Arctic University of Norway, 9038 Tromsø, Norway; karl.o.ottosen@uit.no (K.O.O.);; 2 Division of Substance Use and Mental Health, University Hospital of North Norway, 9291 Tromsø, Norway; 3 Department of Education, ICT and Learning, Østfold University College, 1757 Halden, Norway

**Keywords:** school dropout, family, school, ecological development, social marginalization, peripheral districts

## Abstract

School dropout increases the risk of unemployment, health problems, and disability benefits. Employing an ecological-developmental perspective, we analyzed the interviews of thirteen students from a peripheral Norwegian county, aiming to explore the possible influence of upbringing and schooling on dropout. The analysis revealed that dropout was associated with an unstable family situation, lack of structure in everyday life, unresolved complex learning difficulties, bullying, and a tough existence in a rented room. The participants conveyed a sense of defeat, frustration, and an absence of meaningful alternatives. However, two participants had actively chosen to discontinue their education; this was because they preferred work practice to allow them time to mature and re-orientate in relation to future educational and career choices. Their families and social networks contributed actively to the implementation of their future plans. The findings point to the importance of studying interventions that may prevent school dropout, and that address central factors in the process of school dropout, such as social support, academic achievement, and parental involvement.

## 1. Introduction

School dropout seems to be the result of a dynamic developmental process that starts even before children start school, involving a complex set of individual and environmental factors, such as learning difficulties and personal problems often related to a disadvantaged social background [1,2]. Students who interrupt their education early tend to be disengaged and unmotivated for schooling and often have lower grades and higher rates of absence in their last year at middle school (15 to 16-year-olds). They also feel more often socially excluded or have greater school anxiety than those who complete their education [3]. A high educational level among parents has been shown to be associated with better school completion rates among children [4] and perceived support from both parents and teachers predicts students’ perceptions of control and identification with school, which in turn predicts academic engagement and achievement [5]. Dropping out usually means lower qualifications, which again increases the risk of unemployment, social and health problems, and reduced tax revenues for the state [6,7,8].

Inspired by ecological thinking, Bronfenbrenner [9] launched a new model, ‘the ecological framework’, that focuses on how the social environment directly and indirectly affects the development of children and adolescents. This model describes how layered systems interact with each other and have a particular influence on the child’s psychosocial development. Referring to Mead’s [10] concepts of role construction and ‘significant others’, primarily parents, siblings, friends, and grandparents, Bronfenbrenner [9] argues that the prolonged daily close interaction (proximal processes) of early home life will influence our basic personal, social, and cultural identity. This creates a basis for the internalization of values, codes, and knowledge that will be of significance for subsequent interaction in other environments. Bronfenbrenner et al. [11] metaphorically call this ‘engines of development’. Studies have shown that the structure of the rearing family environment influences children’s cognitive, emotional, and social development. For example, living with single parents and stepparents implies a risk of economic disadvantage, reduced emotional support and supervision, inconsistent disciplining [12], and a greater risk of early dropout [4]. Bronfenbrenner [9] also emphasizes the significance of socialization through daily interpersonal encounters in various other settings, such as at school and during leisure time. For example, the way the teacher communicates educational goals at parents’ meetings and how far parents encourage the learning process at home, will influence the child’s development, school performance, and risk of subsequent dropout [13]. According to ‘the ecological framework’, the development of children and adolescents is also affected by environments where they themselves are rarely or never present, but where their relationships with significant others might be affected. The parents’ employment status (work versus unemployment) and changes in this (layoffs, labor disputes, etc.) may have a negative effect on their children’s lives, in terms of a tighter household budget, poorer work environment, and thus less energy for quality time with the children [14]. Further, political decisions may also play a part. One measure introduced by the Norwegian Government in 1994, called Reform 94, implied fewer courses of study and more hours of theory in vocational courses [15]. The latter is an example of how the political system, through specific curricular adjustments, set new requirements for academic success, which affected students’ performance at school and later educational and career choices. Bronfenbrenner’s ecological-developmental approach can thus form a useful theoretical background for our study of school dropout and encourages consideration of a variety of factors and a holistic perspective.

The northernmost regions of the three Nordic countries Norway, Sweden, and Finland are characterized by vast areas of scattered settlements and long distances to larger population centers, resulting in small and isolated schools and limited job opportunities. In addition, the shift from primarily natural resource-based economies to increased industrial activity during the past three to four decades has led to more centralization, resulting in fewer jobs and increased unemployment rates in rural areas [16]. Many adolescents in these rural areas therefore have to move to get an education and a job. In one north Norwegian county, one-third of the students in the first year of high school in 2010/11 stayed in a rented room [17]. Further, the dropout problem in high school in all three Nordic countries is considerably greater in the northernmost regions [16]. The national average of students in Norway who complete high school within the nominal time is 68.8% [18] with students in general courses having higher completion rates than those in vocational courses. The three northernmost Norwegian counties had the lowest proportion of students completing within the nominal time, at 52.9–63.5% [16]. These large differences between the northern and southern parts of Norway may indicate different causal mechanisms for dropout. There is little research of recent date that focuses on schooling and living conditions of north Norwegian adolescents [19]. The northern Norwegian county councils have called for more research on the dropout phenomenon in the region [17,20].

We designed a qualitative explorative study based on a holistic ecological-developmental perspective [7], to explore how adolescents in one north Norwegian county experienced and understood their drop-out processes in light of their current and past school and life experiences.

## 2. Materials and Methods

### 2.1. Ethics, Consent, and Recruitment

The study adheres to the Declaration of Helsinki [21] and all relevant Norwegian rules and regulations. Prior to starting the study, the study protocol was reviewed and approved by the Northern Norway Regional Committee for Medical and Health Research Ethics.

Following approval from the ethics committee, the study was formally initiated in August of 2010, when information regarding the study was sent to the administrators of all the 17 high schools in the county. Information about the present study was included in the information/invitation letter for a larger study sent to all students starting high school in one of the northernmost counties of Norway in autumn of 2010. This information with a consent form was sent to schools and distributed to students before data collection started in the main study. There was a specific request for consent to be interviewed if the student dropped out. In addition, the information letter and consent form were sent by post to all parents and guardians of students under 16; these parents and guardians had to give separate consent because of the student’s age. Information was also given orally about what participation would entail. Participants could withdraw from the study at any time and have all personal data deleted if desired.

All the data gathered in the study were kept strictly confidential. The audio recordings were only accessible to the researchers and stored in a secure location. Names and other directly identifying information were not included in the transcripts. Only de-identified data in the form of brief excerpts from transcriptions of interviews are presented in the manuscript.

### 2.2. Sample

Those who consented were enrolled in the study and responded to a questionnaire (data from the questionnaire are reported elsewhere) in 2010. Of the 1676 students who agreed to participate in the main study, 1538 students also consented to be interviewed if they dropped out of school. 

Students that had not consented were of course excluded, as were students under the age of 16 where the legal guardians had not consented. In addition, we excluded students that had been granted formal leave from school, for instance due to illness, pregnancy, or maternity leave.

The County’s school administration office had an overview of the students that dropped out of school, and kept the researchers updated on those who also had consented to participate in the study and that were not excluded due to being on formal leave. Altogether, 98 students who had consented to participate in the study left school during their first year of schooling. Interview participants were selected strategically on the basis of gender (at the time of the study only two categories existed in the school database, male and female), place of residence (urban and rural), and subject specialization (academic or vocational).

Based on these criteria, we contacted 23 people (12 girls and 11 boys) by telephone and asked if they would agree to be interviewed. In the end, 13 interviews were conducted with six girls and seven boys, aged 16–21 years, who had studied at five urban and four rural schools. Participants’ dropout occurred between 5 and 18 weeks following school entry and the interviews took place between 15 and 31 weeks after dropout. 

### 2.3. Data Collection and Procedure

The interviews of the students—the data reported on here—were carried out in the period 12 January 2011 to 8 June 2011. The first and second authors conducted individual qualitative interviews lasting from 30 to 80 min. Ten interviews were conducted face to face at a place and time previously agreed upon by telephone and adapted to participants’ wishes. Three interviews took place by telephone. All interviews were audiotaped and transcribed verbatim.

Prior to the interviews, some themes and procedures were established for the interviews and an initial interview-guide was made. As new interviews were carried out, the guide was continuously discussed among the researchers and updated to reflect the knowledge that had been gained so far and to enable new interviews to provide a deeper understanding of the most important and challenging experiences expressed in previous interviews, and to help the researchers decide whether the issues had been elucidated adequately [22].

Because the purpose of the interviews was to elicit information regarding the participants’ experiences and views, a main principle was that the participants were encouraged to talk freely about their experiences and to share their individual stories. The interviews began with an open question: ‘Could you tell me about your experiences from high school and what made you decide to quit?’

It was also important that the person doing the interviews responded appropriately and followed up on the information given by the participants. This meant that the interviews aspired towards a dialogical form more than a question and response format. By summing up what the participants had said and asking clarifying related questions, it was possible to check whether our understanding reflected what the participants wanted to convey. Thus, in the initial part of the interview, the participants were asked to talk about their school experience and any topic the participants believed were important with respect to this experience. Moreover, the participants were asked if they could talk about both positive and negative experiences relating to school and also how these experiences tied in with other aspects of their lives. Our experience was that the participants became more expressive and personal in their descriptions towards the end of the interviews.

At the end of the 13 interviews, the researchers judged that data saturation had been reached. This means that the researchers felt they had obtained a rich dataset that fully addressed the topics of the study and that some repetitive patterns could be identified in the data. The researchers assessed that adding further interviews was not necessary in order to perform the analysis.

### 2.4. Qualitative Analysis

The analysis was performed primarily by the first author. The choice of method should depend on the research objective. Since the purpose of the study was to describe and develop knowledge regarding the experiences and lifeworlds of the participants, a qualitative methodology was deemed most suitable. Qualitative interviews followed by a qualitative analysis allow for an inductive approach, with the development of knowledge and hypotheses. In our case, the topic is the process of high school dropout. We chose the method of systematic text condensation, as described by Malterud [23]. This method is widely used and is inspired by the phenomenological approach of Giorgi, where the goal is to develop new knowledge about people’s experiences and lifeworld within a particular field [24].

The steps were as follows: (a) the transcribed interviews were read and re-read separately in conjunction with notes written during the interview or afterwards, in order to gain a general overview of the material, (b) meaning units were identified, representing different aspects of the participants’ experiences of upbringing and schooling; these were coded and distributed into code groups (not determined a priori), (c) the content of each code group was condensed, and (d) it was then summarized to provide generalized descriptions of participants’ experiences that may have influenced a school career that ended with dropout. The software program NVivo Version 10 [25] was used to extract and collate the meaning units into code groups. The findings were validated by systematically comparing contents and categories with the original material throughout the entire analytical process [23].

## 3. Results

### 3.1. Introduction

A systematic reading and analysis of the transcribed interviews and notes revealed one core category and four sub-categories, which describe in more detail the complex interactions that took place in different socialization arenas from childhood until dropout. The categories were formulated using the participants’ own words. Quotes were used to allow the participants’ own voices to be heard. In Table 1, we present an overview of the core category and the four sub-categories.

### 3.2. Core Category Difficulties in School: ‘I’m Worried about My Future but School Just Makes Me Feel like a Loser.’

All participants except one said that they saw education as important to ensure a good life in the future. But several also acknowledged that learning and concentration problems had led to much frustration over difficult theoretical work which they felt the teachers expected them to do on their own. This was hard to deal with and for many had been an important factor in the decision to drop out of school.

In the data, we found several stories about how uncertain the participants had been in their initial choice of subject areas, and how their experiences from high school made it hard to imagine any kind of education or job that would suit them. One participant elaborated on his deadlock as follows:

‘You make the foundations for your future with an education. That’s important for getting a job, money, security, family, and things like that... I understand the situation’s serious, and I think about it sometimes—it’s a drag. But as far as education goes, I’ve got no idea what to do.’

Many participants said that their difficulties with schoolwork soon became such a huge burden that they had no pleasure in going to school; this then resulted in long periods of absence. Several participants explained that their struggles affected their performance in class, which in turn led to a general sense of dissatisfaction. They were sidelined during the breaks when classmates continued to talk about the subject matter in the canteen. One girl reasoned about her decision to leave in this way:

‘I was very unsure about how things would be afterwards, but I realized I simply couldn’t stand another six months with all those subjects I didn’t like. And as I didn’t have anyone to be with, and every day lasted for ever, when you’re all by yourself in the breaks and so on, well—I felt like I couldn’t stay at school!’

While most participants said they had no job possibilities and acknowledged that they had spent most of their time at home after dropping out, there were two who had got into fulltime jobs with the help of close relatives and their acquaintances. Their decision to leave school early had been taken in consultation with their parents, but only after they had been assured work. Both argued for their decision by saying that they saw no reason to continue their education when learning outcomes were minimal. They expressed a desire to return to school when they were ready to make a more conscious choice about the work they would like to do. While one was confident that work experience would enhance the possibility of completing schooling, the other said:

‘What I do isn’t really that important as long as I like it. At work it’s fine and we get on great together. But the best thing is knowing as much as possible about the job you have to do, and then the ideal thing is to have an education.’

### 3.3. Sub-Category the Importance of Parents: ‘I Maybe Didn’t Have a Structured and Stable Childhood.’

Participants often linked their narratives to home and parents when talking about various school-related problems. There were a number of participants who suggested that their parents did not take the time to help them with their homework, and one participant explained that sheer frustration often led to a noisy argument when neither she nor her parent understood the homework assignments. Another said:

‘I maybe didn’t have a structured and stable childhood. And as for school, I’ve missed having a dad or mum to back me up. Someone who tells you to hurry up and get off to school. If things are working properly in your life, it’s easier to go to school.’

We heard stories of inadequate care, where single parents who were out of work due to substance abuse and mental health problems had left the children to themselves from an early age with the responsibility to get up in time for school in the mornings. One participant described in detail how she periodically had to take care of her younger brothers and sisters because of the mental disorder of her single parent and the lack of help from other relatives and the professional network. This led to a high rate of absence from school, concentration problems, and major concerns for a little sister who was bullied at school. It was difficult to catch up with the lessons, which led to further problems in high school. Others had experienced violence and quarrelling between parents, ending in divorce and later disagreement about the rules for access and financial support. This led to participants distancing themselves from one of their parents. One participant said:

‘I have no contact with my biological father, because he’s, excuse the expression, a load of shit...When we were young, he never looked after us. He was kind of never there...I’ve got a mother who’s helped me a lot through the years.’

For some participants, domestic problems meant that they had to move repeatedly during their upbringing, giving them a sense of rootlessness and a need to belong somewhere. Several said they had trouble in talking about difficult situations and having to comply with different rules, as one explained:

‘I moved back and forth between mum and dad and grandma...At mum’s house, I had loads of freedom...I had lots of nightmares when I moved to live with dad who was very strict...it was really, really hard to cope with that, when I’d always been used to doing almost as I pleased at mum’s house.’

### 3.4. Sub-Category the Importance of Teachers: ‘I Wish the Teachers Could Have Sat down at My Desk and Gone through the Maths with Me.’

We heard stories from primary and middle school about how difficulties in concentrating and understanding the content of textbooks easily led to frustration. Some found it problematical to ask for help after the teacher had gone through the lesson on the blackboard, when everything in the textbook was difficult and they did not quite know how to explain which part they did not understand. It could end in conflict with teachers who neither understood what they were struggling with nor gave them sufficient help. Some said that learning difficulties had sometimes been used against them. One girl who had not been diagnosed with dyslexia until the sixth grade said:

‘I remember a teacher who came up to me and said, I don’t think you’ve got dyslexia, I think it’s more like you can’t be bothered to do your best.’

The majority of the participants said they had expected more practical sessions in high school to enable them to use their capabilities and talent on the kind of work they might expect to meet in their future job. Many expressed frustration at having to continue the impossible struggle with complicated theoretical subjects, and this also soured their relationship with teachers right from the start of high school. A boy who maintained he did not know English got into a conflict and eventually felt he was being harassed by the teacher who constantly pressured him to read aloud in class. In a reconciliation meeting the teacher apologized, but there was no further discussion of his specific learning difficulties. In the next lesson he was given ten short English books to replace the book on the syllabus. But he found it all pointless; he made no progress, and it was difficult to keep track of all the books. Another participant recalled:

‘I told two maths teachers that I was struggling with maths and why I found it hard. The reason was that I had not had it for a long time, and it was difficult to understand and concentrate. I asked for some extra help, but I didn’t get it...I actually think all I wanted was for the teachers to sit down at my desk when the others in the class didn’t need help and go through the maths with me.’

### 3.5. Sub-Category Being Bullied: ‘I Feel like Years of Being Bullied Has Done Psychological Harm to Me.’

Several participants said that they had often felt ignored and excluded by classmates in primary/middle school. Usually, the most popular and physically strong students laid down the rules in the playground. If you were not friends with the leaders, could not afford the latest clothes, or had different tastes in clothes or music, you would soon be squeezed out and end up walking around by yourself. The worst cases involved severe harassment, spitting, and physical violence. Participants mentioned how such ostracism could also spread into the classroom, where the clever and talented students had precedence, being the ones seen and heard by the teachers. At the beginning of high school, some boys had difficulty in getting accepted and making friends in the class after false rumors had been spread about them at school. Several of our participants described in detail how they could not shake off the unpleasant feeling of being picked on; this led to much absenteeism due to school anxiety and difficulty concentrating in class. One participant explained it in this way:

‘I’ve become a very asocial person...If I include all the years, I’ve been bullied for over six years. When I go to school, I just switch off everything. Then it’s just a body sitting there in class...when people say something to me, I snap back at them straight away. I feel like school has done psychological harm to me; I’ve been permanently damaged by all the years of bullying.’

Several participants were bad at sports and since they did not like competing either, they were always chosen last for the team. One explained how failure on the football pitch evolved into ‘not being socially accepted’:

‘I played football until sixth grade, but there I eventually almost got hated out of the team, because I wasn’t super interested in football...the others soon got much better than me. I was the worst one in the team. Many of the others got annoyed about it, so they picked on me a lot.’

### 3.6. Sub-Category Being Alone: ‘Living Alone in a Room Kind of Didn’t Work Out.’’

Living in a rented room by oneself was an extra burden. It had been difficult to find somewhere to stay at the school. It was hard to budget enough money for both rent and food. The grant was small, and parents often had little extra money to contribute. One girl who had shared a cramped little bedsit with a female friend found it tiresome to have to concentrate on her homework, so she often chose not to do it. The two girls would argue about who was to do the cooking and all the other practical things involved in living away from home. Our participants said they felt sad and depressed and missed having adults to talk to about homework and other things that bothered them:

‘I was really homesick. It’s not easy to live 500 miles away from your mum...My contact teacher didn’t like me, but there was another teacher who cared a lot. I could ask him for help if there was anything I didn’t understand or that was bothering me, as of course mum wasn’t there.’

Some of those who had lived in rented rooms said they had grown up in villages and were unused to relating to what big towns could offer both day and night. They admitted that this had made it more difficult to organize their lives and have good routines in separating school from leisure time. New friends in the new town also had an influence on their education. One participant said:

‘Living alone in a room kind of didn’t work out. You got your head full of other things than going to school. It was more fun to walk around town, go shopping and that, go out drinking with your friends. That was what kind of messed things up a lot.’

## 4. Discussion

With reference to an ecological-developmental perspective, we wanted to explore the significance of the experienced school and home environment for students’ decisions to drop out of high school in a peripheral county in northern Norway. The analysis gave rise to a core category, which summarized the informants’ perception of their encounter with high school, and four sub-categories that convey in more detail the diversity of interacting factors in the home and school environment that may have affected the students’ development and socialization in relation to dropout. Below, we discuss our findings and their implications.

Firstly, a central theme in the material was frustration at having failed at high school and the resulting fear of losing access to employment and a financially secure future. Our analysis revealed that most had complex learning difficulties which had been given little attention or remedial help in their previous schools, but which in the transition to high school could no longer be compensated for. This academic underachievement then affected socialization with classmates, leaving the participants with a sense of being left out of friendship groups. When the interviewees began to talk about this deadlocked and frustrating situation, they expressed a need to probe more deeply into all the problems they had been struggling with through much of their education as an explanation for dropping out of school. Our participants’ descriptions correspond well with frameworks and models previously described [1,3], where dropout is portrayed as a process that begins early. These studies show that complex learning difficulties, a low skill level, knowledge gaps, and a stressful environment are common obstacles to students’ ability to meet the increased demands for academic skills, independence, and social skills during the integration process in high school. Our findings indicate that the combination of failing to reach the academic level required to follow the lessons and meet curriculum requirements and the simultaneous experience of being excluded by classmates can undermine the sense of control and autonomy necessary for the mastery of important developmental tasks in late adolescence [26]. This can lead the student into a ‘negative developmental cycle’ [1], which involves the loss of self-esteem and the need to withdraw from the school environment, manifested in our study by high absenteeism and eventually dropout as the endpoint of a long process that began early in the student’s school career. After dropping out, many participants had not found a job and spent most of their time at home. For many adolescents, this situation can be the starting point of a marginalization process [27], which increases the risk of various serious social and health problems [2]. However, in our study this was not the situation for all the participants. Interestingly, there were two participants who, in consultation with their parents, temporarily rejected education in favour of practical work experience. Their plan was to return to school when they were older and had more experience, to enable them to make a more mature, well-considered, and sound educational choice. While this practical work experience may be a good solution for some students, Polesel et al. [28] state that this may work poorly for others. It has been documented that work training cannot easily remedy the learning disabilities that many of these young people are struggling with. A further factor is the students’ often inadequate ability to relate to the fixed schedules and routines of the workplace [28]. However, experiments with combined practical and theoretical training programs, in that order, have been shown to work best for marginal student populations [29]. Social support is an important factor in well-being and mental health in general [30], and the re-enrollment of students that have dropped out of school or work may rely in part on increasing social support [31].

Secondly, given the central role of parents as significant others, as emphasized by Bronfenbrenner [9], it is highly probable that with insufficient monitoring and help at home to provide a framework and structure for homework and schoolwork, as experienced by our participants, adolescents with limited basic ability to master school requirements can easily become frustrated and thus not identify with school culture—with a consequent low commitment to schooling [5]. It has been shown that there is an increased risk of early school dropout among children growing up in homes and environments without a culture of academic learning; this is generally related to a low parental educational level, a low level of school-related help at home, and a low level of educational aspirations on behalf of the children [2]. Conversely, it has been demonstrated that children are protected from dropout by home environments where parents, irrespective of income and ethnicity, become involved and allocate sufficient time to help with homework and lend support to their child’s education [32,33]. In our material, conflictual post-divorce relationships between parents, with disputed parental responsibility and divergent views on parenting and monitoring of schoolwork, seem to have led to poor quality and continuity in the pupils’ work. This emerged in stories about all the practical and school work-related difficulties that arose and increased over time through constantly having to comply with new rules, such as homework time, indoor time, and outdoor time, when moving back and forth between the mother’s and father’s homes. This could partly explain why parental divorce may have a negative effect on children’s capacity to adjust to academic challenges; furthermore, academic problems also seem to increase with age [34]. Moreover, an upbringing in a dysfunctional family, typified by gross neglect because of parental alcohol or drug abuse and/or mental problems, led to some early leavers in our study moving with one parent to another part of the country. It has been found that students who change residences and/or schools have an increasing risk of dropping out of high school [35], especially students with lower socioeconomic status, problem behavior, and high absenteeism [4]. The participants in our study with similar backgrounds felt that the necessity to live in council housing in a tough socially deprived neighborhood was an additional burden that created uncertainty, insomnia, anxiety/depression, and worries about the future.

Thirdly, our analysis revealed a pattern of participants with unresolved complex learning difficulties such as inadequate basic skills in reading, writing, and mathematics, which, through the school years, had also limited learning in other subjects. The data were strikingly characterized by scant constructive interaction between teacher and student in classroom situations where learning difficulties stood in the way of new learning and insight. Considering the fact that many of our participants received little school-related support and help at home, such a relationship with teachers may have increased their frustration at their own shortcomings and contributed to the early dropout. We find support for this in Jimerson et al. [36] who state, ‘The capacity for such students to complete school and achieve good academic outcomes is strongly associated with the quality of the experience at school, particularly the nature of the student-teacher relationships which exist.’ (p. 32). Jimerson et al. found that students who experienced a low quality of the teacher-student relationship also showed low emotional commitment to schooling [36]. This is in accordance with Bergin and Bergin [37], who argue that one of the preconditions for children in primary school and teenagers in high school to be successful is the establishment of a secure relationship between teacher and student, leading to a greater desire for learning. A secure learning climate where the learner is seen and acknowledged by the teacher can make it easier for students to realize and articulate their learning difficulties. A competent teacher therefore has a better basis for adapting the teaching and giving constructive feedback that promotes positive mastery experiences and social development [37,38,39].

Fourthly, our analysis showed that almost half of our participants had experienced being excluded and/or physically or verbally harassed in school as well as in extra-curricular activities outside school hours, in these often small outlying communities. This is in line with Nordhagen et al. [40], who have documented that Nordic students from low-income families and with single parents with low education, as was seen in many of our participants, run a greater risk of being bullied. From an ecological-developmental perspective, our participants’ experiences of being subjected to repeated systematic and comprehensive harassment, shoving, and ostracism over time by dominant classmates in middle school may have reduced their ability to develop the social skills necessary to be accepted and included in the new environment of high school. When former victims therefore found that ostracism and bullying took new forms in high school, this may partly have been due to the victims’ insufficient development of certain skills such as the ability to cooperate and demonstrate empathy, good assertiveness, responsibility, and self-control in encounters with peers. Our assumptions correspond with findings from other studies showing that bullied students had fewer friends, were more introverted, and generally lacked social skills, in comparison with classmates not exposed to bullying [41]. This underlines the importance of creating sound and stable psychosocial learning environments with a tolerance for diversity, where vulnerable students can experience security, inclusion, acknowledgement, and personal development. When bullying is not captured or alleviated by adults at school or home, the result may be psychosomatic complaints, such as stomach pain and headaches, along with high absenteeism due to school anxiety as reported by our participants. This corresponds with findings in a previous Nordic study [40]. Further, missed lessons and reduced effort by these students may be expected to hamper academic progress and lead to poor grades, as was the case for most of the victims in our study. We find support for our reasoning in a Norwegian study [42], which shows that a bullying environment at school leads to a general sense of insecurity among students, which in turn can impair performance. According to Strøm et al. [42], students attending schools where bullying was prevalent averaged almost a full grade lower than students in schools with little bullying.

Fifthly, we found that participants who had to move to a rented room in the transition from middle to high school had adjustment problems of both a practical and psychosocial nature, such as an inadequate organization of chores, cramped conditions, and disturbing interference from their roommates. This led to general dissatisfaction, which, coupled with a lack of monitoring and help with schoolwork, slowed their academic progress. The frustration expressed by the participants at once again being a school failure, together with the extra burden of living in a room away from home, may partly explain both the increased risk of dropout found among students in bedsits [17] and why two of the three northernmost counties top the dropout statistics in Norway [18]. We also found that our bedsit participants expressed a strong desire for increased monitoring and contact with responsible adults connected to the school, which they believed would have alleviated some of the difficulties of living away from home. In another study from the same region, Lie et al. [20] reported that dropout was reduced by external educational measures where students lived with host families or in a residence hall manned around the clock by social workers. One of the success factors highlighted in this study was the close collaboration of social workers with schoolteachers and counsellors for students living away from home, as well as the close monitoring of these students after school hours along with enjoyable activities linked to the local authority’s leisure program. Further, limited finances as a result of the poor economic situation of unemployed and single parents, combined with small grants and high rent, were a considerable additional burden for those living away from home. This corresponds with other studies which show that children from disadvantaged households are more likely to drop out of school [2,27]. Our study also revealed the difficulties involved in balancing school and leisure time for bedsit students. This often ended with students opting out of school in favour of other more pleasurable activities like going shopping with other students. These were often unmotivated students who did not see the point of putting much effort into schoolwork. This suggests that a contributory factor to school dropout may be the mutual influence of friends within networks, which in our study were described as relatively loose and superficial, involving activities incompatible with school and which increased absenteeism. Our assumption is supported by the findings of an American study [35], where high school students who moved and/or changed schools were twice as likely to drop out of school as non-mobile students. The differences between the mobile and non-mobile students could best be explained by the structure and composition of their networks of friends, where mobile students tended to have smaller, narrower networks and a less central position within the networks. Students who had friends with lower grades and were also more peripheral in their networks were more likely to drop out.

This study has some limitations. The fact that the interviews were performed in 2011 could have implications for the application of the findings. While this data was collected some years ago, we believe it is still highly relevant as the problem of school dropout remains. Unfortunately, on a structural level, relatively little has been done in Norway in the last decade to address the issues brought up in our study. As the recent statistics on school dropout show (mentioned in the Introduction), this problem remains significant [16]. We therefore believe that the stories told by the participants about why they dropped out of school remain highly relevant today.

As in most qualitative research, the study sample was small, which renders generalization difficult. In any semi-structured interview, the interaction between interviewer and interviewee will affect the quality of the interview. When describing their situation, the participants may have consciously or unconsciously sought to respond as they believed the interviewer expected; this was how they reacted to familiar school tests with right and wrong answers. On the other hand, according to Bronfenbrenner’s theory [9], for this kind of ecological study, the ecological validity is strengthened by the interviews being conducted outside the school context and often close to the home where the participants lived and had grown up. However, to reduce bias, a continuous, conscientious, open, and listening attitude combined with reflexivity was strived for [23]. Our impression was that the participants openly shared their experiences and perceptions of dropout-related factors. But we may assume that those who refused to be interviewed probably would have had greater difficulty in communicating their situation than those who consented to interviews. Critical reflection and discussion in the research group throughout the entire process, especially in the systematic analysis of the interviews, helped to create the necessary distance required to grasp the essence of the participants’ communication [23].

There is clearly a need for interventions that address the issues we have described in this study. One example of such an intervention, that involves the parents and that focuses on basic academic skills in a socially supportive group setting, is the Boy’s Camp [38]. This intervention consists of a two-week learning camp run by socially and academically proficient adults, followed by regular mentor group meetings. Informing and involving the parents is a central part of the intervention. Much emphasis is placed on learning to learn, and to systematically work on reading, writing, and maths. In addition, the participants are encouraged to adopt positive habits, including sleeping and eating in a healthy manner. Preliminary research [43] suggests the intervention may have positive effects, but more research is needed.

## 5. Conclusions

With its focus on experiences of upbringing and schooling in a peripheral county in northern Norway as described by the interviewees, this study revealed a variety of factors that separately and in combination may have contributed to a school career that culminated in dropout. We saw the outlines of a pattern repeated in the narratives, which to some extent was consistent with international research, where dropout is described as a process that begins at an early age. This means that many had negative experiences from everyday school-related interaction in ‘proximal processes’, such as lack of help for complex learning difficulties at primary school and for homework at home, and a draining of energy in protecting themselves from playground bullying, leading to poor concentration and academic performance. This could indicate a need for better home–school collaboration and an increased focus on early assessment and adapted education for students with learning difficulties. Further, many participants had a home background with low socio-economic status and experiences of dysfunctional family relationships, leading to discontinuity in childhood in terms of instability, changes, and repeated moving. They had never felt integrated into the school environment in these often small and sparsely populated communities and felt that this inhibited social integration in other social arenas dominated by teachers and students from their school. When such circumstances affect ‘the engines of development’ or development of identity in childhood at home and/or school, the result may be low self-esteem and lack of self-efficacy. Here may lie the source of the participants’ frustration at the academic and social challenges that were more demanding than they had expected to find at high school. Moving to a new place and living in a rented room seem to be an extra burden for some participants, who already were in a marginal position in their local communities and school environment. Since many also seem to have difficulty in structuring time for school, homework, and leisure, this indicates the need for a follow-up system in the transition to high school. However, participants who had been recruited to work through their parents’ network were well integrated in the local community. This implies that within the parents’/families’ social position, network and/or role as ‘significant others’, there lies a potential to find alternative pathways to succeed in school/work. There is a need to implement and study interventions that address school dropout [44], including interventions that focus on the central issues described in the present study, such as social support, academic skills, and parental involvement.

## Figures and Tables

**Table 1:** Overview of categories.

Core category *Difficulties in school: ‘I’m worried about my future but school just makes me feel like a loser’*
*Sub-category* *The importance of parents: ‘I maybe didn’t have a structured and stable childhood’*
*Sub-category* *The importance of teachers: I wish the teachers could have sat down at my desk and gone through the maths with me*
*Sub-category* *Being bullied: I feel like years of being bullied has done psychological harm to me*
*Sub-category* *Being alone: Living alone in a room kind of didn’t work out*

## Data Availability

The interview data on which the study is based is not publicly available due to privacy regulations.
